# Comprehensive Quantification of (Poly)phenols in Lotus japonicus with and without Arbuscular Mycorrhizal Symbiosis

**DOI:** 10.1021/acs.jafc.5c02061

**Published:** 2025-05-26

**Authors:** Josef L. Ranner, Georg Stabl, Cindy Martyniak, Michael Paries, Andrea Spaccasassi, Caroline Gutjahr, Timo D. Stark, Corinna Dawid

**Affiliations:** 1 Chair of Food Chemistry and Molecular Sensory Science, TUM School of Life Sciences, 9184 Technical University of Munich (TUM), Lise-Meitner-Str. 34, Freising 85354, Germany; 2 28322 Max Planck Institute of Molecular Plant Physiology, Am Mühlenberg 1, Golm, Potsdam 14476, Germany; 3 Plant Genetics, TUM School of Life Sciences, 9184 Technical University of Munich (TUM), Emil-Ramann-Str. 4, Freising 85354, Germany; 4 TUM CREATE, 1 CREATE Way, #10-02 CREATE Tower, 138602 Singapore; 5 Functional Phytometabolomics, TUM School of Life Sciences, 9184 Technical University of Munich (TUM), Lise-Meitner-Str. 34, Freising 85354, Germany

**Keywords:** arbuscular mycorrhiza, flavonoids, *Lotus japonicus*, plant polyphenols, quantification method, symbiosis, UHPLC−MS/MS

## Abstract

In the present study, a highly specific, accurate, and robust ultrahigh-performance liquid chromatography–tandem mass spectrometry (UHPLC–MS/MS) method for the simultaneous quantification of 50 plant (poly)­phenol analytes was developed and validated to assess the effect of arbuscular mycorrhizal (AM) symbiosis on the (poly)­phenolic content of the model legume Lotus japonicus (L. japonicus). Determination of molar concentrations of analytes in roots and shoots of wild-type and AM mutant L. japonicus (with and without AM symbiosis, respectively) revealed an overall increase in (poly)­phenols in mycorrhizal plants. Time-course observation over 10 weeks showed a shift in (poly)­phenol concentrations, especially in the roots. In total, 13 analytes were notably more abundant in young AM roots, suggesting a potential role in symbiosis initiation. An accumulation of various (poly)­phenols at later stages of symbiosis might indicate a potential involvement in arbuscule degradation or AM autoregulation.

## Graphical abstract

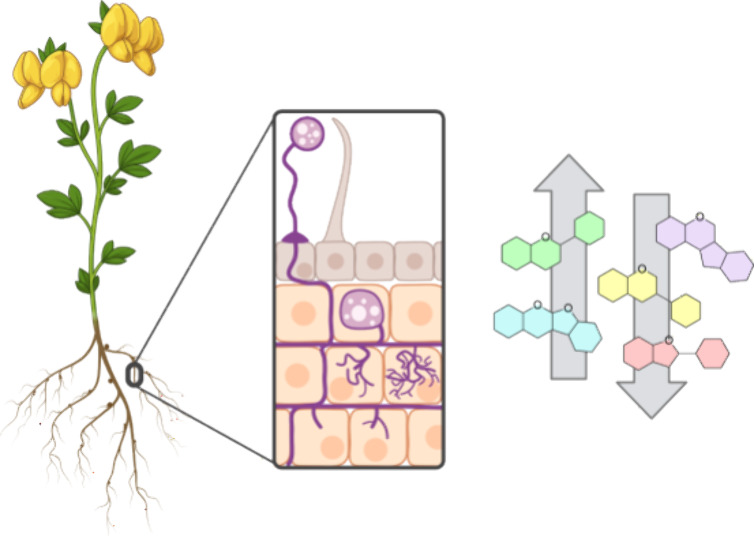

## Introduction

Legumes play a crucial role in global nutrition owing to their high content of protein, fiber, minerals, vitamins, and polyphenols, which exhibit antioxidative properties and health-protective effects.^1^ Plant-based protein sources such as legumes offer a sustainable dietary solution to combat climate change by mitigating global warming and reducing greenhouse gas emissions associated with livestock production.^2^ Root–microbe symbioses such as arbuscular mycorrhiza (AM)—a symbiosis with Glomeromycotina fungi (arbuscular mycorrhiza fungi, AMF)—enhance the growth and yield of legumes.^3–8^ These symbiotic relationships have been investigated using model legumes such as Lotus japonicus (L. japonicus) or Medicago truncatula as they are diploid and in part easier to cultivate than other crop legumes, facilitating the accelerated acquisition of crucial insights into AM regulation and development. With its small, fully sequenced genome of 450 Mbp, a generation time of 3–4 months, and self-fertility, L. japonicus is an ideal model plant.^9–12^

In AM symbiosis, the fungus provides mineral nutrients such as phosphate and ammonium, obtained from the surrounding soil through its extraradical hyphae. The host plant reciprocates by supplying photoassimilates to the fungus.^13^ Symbiosis initiation requires structural and functional modifications of host root epidermal and cortical cells and a significant level of cellular, genetic, and molecular regulation by both symbionts.^14^ Prior to root colonization, chemical signals are released to facilitate mutual recognition. Plant-derived strigolactones promote fungal hyphal growth and branching,^15^ whereas fungal Myc factors enhance symbiosis signaling and suppress immune signaling in the host plant.^16^ Fungal hyphae penetrate the outer root cell layers, expand within the cortical apoplast, and invade inner cortical cells, forming arbuscules. These highly branched structures are surrounded by the periarbuscular membrane, which is required for nutrient exchange through phosphate and ammonium transporter proteins such as PT4 and members of the AMT2 family.^14,17–19^

In a previous study, we demonstrated that AM symbiosis causes an accumulation of (poly)­phenolics in L. japonicus roots at 7 and 10 weeks after inoculation with Rhizophagus irregularis (R. irregularis) spores.^20^ The previously unknown lotuschromone (**42**), lotusaldehyde (**48**), and lotuscarpene (**35**) were isolated from L. japonicus roots.^20^ Other flavonoids (aglycones) and isoflavonoids that were identified in L. japonicus include isoliquiritigenin (**31**),^21,22^ epicatechin (**2**), epigallocatechin,^23^ gossypetin, isorhamnetin (**25**), kaempferol (**19**), myricetin (**6**), quercetin (**12**),^24^ isosativan (**47**),^25^ sativan (**44**),^21,26^ vestitol (**33**),^21,26–32^ daidzein (**9**), formononetin (**32**),^21^ and medicarpin (**36**).^21,28,29,33^

Polyphenols are a molecular compound class of secondary metabolites containing two or more aromatically bound hydroxy groups and derivatives thereof.^34^ Biosynthesis of the polyphenol subclasses phenolic acids, stilbenes, flavan derivatives, and lignans begins with phenylalanine formation via the shikimate pathway.^35,34^ Phenylalanine is converted to *p*-coumaroyl-CoA and hydroxycinnamic acids (phenylpropanoids)^36,37^ or to naringenin chalcone, which is converted to naringenin (**21**), which enters the flavonoid pathway.^34^ *p*-Coumaroyl-CoA is alternatively converted to isoliquiritigenin (**31**) and liquiritigenin (**11**) and subsequently, via 2,7,4′-trihydroxyisoflavanone, to the isoflavonoid daidzein (**9**).^38^ Isoflavonoids have their B-ring attached at the C3 instead of the C2 position of the C-ring and are predominantly formed in legumes.^39–41^

Polyphenols possess antioxidant and UV-absorbing activities that render them crucial protectants against reactive oxygen species (ROS) and DNA-damaging UV-B radiation.^21,36,42^ Furthermore, polyphenols and especially flavonoids often exhibit phytoalexin properties, playing a significant role in plant defense against microbial pathogens, fungal pathogens, and herbivores such as insects.^43,44^ In addition to acting as allelopathic agents when released into the soil,^45,46^ polyphenols facilitate plant communication with beneficial microorganisms in the rhizosphere.

Notably, some flavonoids induce the expression of *nod* genes of nitrogen-fixing rhizobia, which form root nodule symbiosis with legumes, and the overall polyphenol profile of L. japonicus roots is severely altered during root nodule symbiosis.^47–50^ In AM symbiosis, flavonoids stimulate mycorrhizal spore germination and promote hyphal branching.^51^ AM symbiosis induces flavonoid biosynthesis, and the overall flavonoid content of mycorrhizal roots is generally higher than that of nonmycorrhizal roots in different plant species.^52–57^ In another model legume, Medicago truncatula, the levels of certain isoflavonoids, including daidzein (**9**), ononin, and malonylononin, were increased during the later stages of fungal development, as opposed to control plants.^58^ Apigenin (**18**), naringenin (**21**), hesperetin (**26**), quercetin (**12**), quercetin-3-*O*-galactoside, 7,4′-dihydroxyflavone, 7,4′-dihydroxyflavanone, and other flavonoids stimulate AMF spore germination and hyphal branching.^59–62^ Conflicting effects have been reported for some isoflavonoids such as biochanin A (**40**) and formononetin (**32**), with a stimulatory effect on mycorrhizal root colonization by Glomus fasciculatum and Glomus intraradices (syn. R. irregularis)^63–65^ and an inhibitory effect on the growth of Glomus fistulosum and Gigaspora margarita.^59,66–68^ Some flavonoids including acacetin (**38**) and rhamnetin have been reported to inhibit hyphal growth and branching of some *Gigaspora* and *Glomus* species.^60,62^ Additionally, the coumaronochromones lupinalbin A (**28**) and ayamenin D (**29**), pterocarp-6a-ene lotuscarpene (**35**), pterocarpan medicarpin (**36**), chalcone isoliquiritigenin (**31**), isoflavonoids daidzein (**9**), genistein (**17**), 2′-hydroxygenistein (**10**), biochanin A (**40**), and sativan (**44**), and flavonoid liquiritigenin (**11**) significantly inhibited spore germination of R. irregularis.^20^ These findings suggest that flavonoids, isoflavonoids, and related compounds are involved in the regulation of root colonization in a highly specific manner dependent on the flavonoid, involved fungal and plant species, and the developmental stage.^51,56^

The accurate determination of (potentially) bioactive polyphenols is crucial to expanding the understanding of plant response to AM symbiosis at the molecular level. As reported in previous studies,^21,24,27,30^ LC–MS is an effective tool for polyphenol analysis in crude extracts of L. japonicus tissues with high accuracy, sensitivity, and selectivity. The present study aimed to establish and validate a rapid, high-throughput ultrahigh-performance liquid chromatography–electrospray ionization–tandem mass spectrometry (UHPLC–ESI–MS/MS) method for the simultaneous quantification of 50 polyphenolics and phenolics in L. japonicus extracts. The proposed method was used to determine analyte concentrations in various L. japonicus mutants, at different harvesting times, and in aboveground and belowground tissues (shoots and roots, respectively). To the best of our knowledge, this is the first study to comprehensively assess (poly)­phenol levels in L. japonicus under the influence of arbuscular mycorrhiza.

## Materials and Methods

### Chemicals

The following chemicals and solvents were purchased commercially: acetonitrile, methanol (LC–MS grade, Honeywell, Seelze, Germany), luteone (**43**) (AchemBlock, Hayward, CA, USA), 7-hydroxy-5,4′-dimethoxyisoflavone (**27**), glyurallin A (**50**, internal standard [IS]) (Ambinter, Orléans, France), 2′-hydroxygenistein (**10**) (BioCrick, Sichuan, PRC), demethylwedelolactone (**4**, IS), wedelolactone (**15**), genistein (**17**), (−)-maackiain (**34**), 7,3′,4′-trimethylluteolin (**46**), licoisoflavone B (**52**) (Biorbyt, Cambridge, UK), sophoricoside (**5**), thevetiaflavone (**7**, IS), 2′-hydroxyformononetin (**22**), (−)-vestitol (**33**), (−)-medicarpin (**36**), (−)-glyceollin I (**39**), (−)-phaseollin (**41**), (−)-sativan (**44**), salvigenin (**45**), (−)-isosativan (**47**), 5-hydroxy-7,4′-dimethoxyisoflavone (**54**), lupinalbin B (**55**), 3,7,4′-trimethylkaempferol (**56**, IS), alpinumisoflavone (**57**, IS) (Biosynth Carbosynth, Compton, UK), coumestrol (**16**) (Enzo Clinical Laboratories, Farmingdale, NY, USA), (+)-catechin (**1**), genistin (**3**), myricetin (**6**), quercetin (**12**), apigenin (**18**), kaempferol (**19**), (−)-naringenin (**21**), syringetin (**24**), isorhamnetin (**25**), (−)-hesperetin (**26**) (Extrasynthese, Genay, France), moracin M (**8**, IS) (MedChemExpress, Monmouth Junction, NJ, USA), 5-hydroxy-7,4′-dimethoxyflavone (**51**) (PhytoLab, Vestenbergsgreuth, Germany), acetic acid, potassium hydroxide, (−)-epicatechin (**2**), daidzein (**9**), (−)-liquiritigenin (**11**), luteolin (**13**, IS), calycosin (**14**), cladrin (**23**), mosloflavone (**30**), isoliquiritigenin (**31**), formononetin (**32**), genkwanin (**37**), acacetin (**38**), biochanin A (**40**), 7,12-dimethoxycoumestan (**53**) (Sigma-Aldrich, Steinheim, Germany), (−)-vestitone (**20**), wighteone (**49**) (Toronto Research Chemicals, Toronto, Canada), and formic acid (LC–MS grade, VWR, Leuven, Belgium). Lupinalbin A (**28**), ayamenin D (**29**), lotuscarpene (**35**), lotuschromone (**42**), and lotusaldehyde (**48**) were isolated as reported recently.^20^ Water used for chromatography was purified using a Milli-Q Reference A+ combined with an Elix Essential 3 water system equipped with a 0.2 μm PES high-flux capsule filter (Millipore, Schwalbach, Germany). Deuterated dimethyl sulfoxide-*d*_6_ (DMSO-*d*_6_) for quantitative ^1^H nuclear magnetic resonance (qHNMR) spectroscopy was purchased from Sigma-Aldrich (Steinheim, Germany).

### Plant Material and Quantification of Root Colonization

Lotus japonicus wild-type (WT) (ecotype Gifu B-129) plants and two allelic mutants each of ccamk (*ccamk-3*, *ccamk-13*), *cyclops* (*cyclops-3*, *cyclops-4*), ram 1 (*ram1-3*, *ram1-4*), and ram2 (*ram2-1*, and *ram2-2*) as well as L. japonicus wild-type plants for monitoring the time course of the AM symbiosis were cultivated as recently reported.^20,69–73^

The seeds were first subjected to scarification with sandpaper for 5 min and then surface-sterilized with 2% NaClO and 0.1% sodium dodecyl sulfate (SDS, Colgate-Palmolive, New York City, NY, USA) in water for 15 min. The seeds were washed three times with sterile water and incubated in sterile water for 3 h. The imbibed seeds were transferred to plates containing 0.8% agar (Duchefa, Haarlem, Netherlands) and incubated at 24 °C in the dark for 3 days to allow germination. For the following 11 days, a light/dark cycle (16/8 h) at 24 °C was applied. The seedlings were cultured in pots containing autoclaved quartz sand (0.7–1.2 mm; Casafino, Munich, Germany) and 40 mL of half-strength Hoagland medium.^74,73^ AM plants were inoculated with 500 spores of R. irregularis (Agronutrition, Toulouse, France) per plant. The plants were watered twice per week with 30 mL of sterilized water per pot and fertilized once per week with 30 mL of half-strength Hoagland solution per pot. The plants were grown in a phytochamber set at 22 °C in a long-day photoperiod (16 h light/8 h dark) with a relative humidity of 60%. Three inoculated pots and three control pots of wild-type and mutant lines were harvested 7 and 10 weeks post inoculation (wpi), respectively, frozen in liquid nitrogen (−196 °C), and stored at –80 °C. Plants from one pot were pooled to represent one biological replicate. One set of wild-type plants was cultivated to monitor AM symbiosis over time. Three pots of inoculated plants and three pots of control plants were harvested at 2, 4, 6, 8, and 10 wpi, and the roots were separated from the aboveground organs (shoots). AM roots and shoots were frozen separately in liquid nitrogen (−196 °C) and stored at – 80 °C. For the control samples, three roots or shoots each from the same pot were pooled to represent one biological replicate.

To determine the root length colonization of AM roots, two or three root systems per pot were boiled (10% KOH in water) for 15 min and washed once with acetic acid (10% in water). The roots were stained using a solution of black ink (5%) in acetic acid (5% in water) at 95 °C for 5 min, washed three times with water, and destained using acetic acid (5% in water), as reported previously.^73,75^ One centimeter-long root segments were analyzed at 10-fold magnification using a light microscope type 020-518500 DM/LS (Leica, Wetzlar, Germany) with a modified gridline intersect method.^76^

### Quantitative ^1^H Nuclear Magnetic Resonance Spectroscopy (qHNMR) to Determine the Accurate Concentrations of Standard Stock Solutions

To determine purity and concentration, each reference compound and internal standard (**1**–**57**; 1–3 mg) were dissolved in deuterated DMSO-*d*_6_ (1000 μL), and an aliquot (600 μL) was transferred to NMR tubes (4.97 × 177.8 mm, Bruker, Rheinstetten, Germany). The qHNMR experiments were performed on a 400 MHz Avance III spectrometer (Bruker) equipped with a 5 mm BBI probe head (Bruker) operated at 25 °C. To accurately quantify the concentration of each reference stock solution, the ERETIC 2 procedure based on the PULCON methodology was used, as previously reported.^77^ The spectrometer was calibrated via an external standard solution containing l-tyrosine (4.34 mmol/L in D_2_O + DCl, 9:1, v:v) using the proton resonance signal at 7.11 ppm (m, 2H) for integration. Data were acquired, and concentrations were calculated using TopSpin (v 3.6.0, Bruker). The stock solutions were stored at −20 °C until further use.

### Internal Standard Stock Solution

For accurate quantification of each analyte using LC–MS, an IS sharing basic structural features was chosen. A list of assigned IS solutions is given in Table S3 in the Supporting Information. Acetonitrile extracts (2 mL) of L. japonicus roots and shoots (50 mg, lyophilized) were analyzed using UHPLC–MS/MS to ensure that those were free of the analytes designated as IS. Single IS stock solutions were combined to obtain an IS stock solution containing demethylwedelolactone (**4**, 125 μmol/L), thevetiaflavone (**7**, 125 μmol/L), moracin M (**8**, 125 μmol/L), luteolin (**13**, 125 μmol/L), glyurallin A (**50**, 125 μmol/L), 3,7,4′-trimethylkaempferol (**56**, 125 μmol/L), and alpinumisoflavone (**57**, 50 μmol/L) in a mixture of acetonitrile and DMSO-*d*_6_ (76:24, v:v).

### Sample Preparation

The roots and shoots (for sample sizes, see Table S2 in the Supporting Information) were lyophilized (Delta 1-24 LSC, Christ, Osterode, Germany), and acetonitrile (1990 μL) and the internal standard stock solution (10 μL) were added to each sample. Homogenization and extraction were performed using a Precellys Evolution tissue homogenizer (Bertin Technologies, Montigny-le-Bretonneux, France) at 6000 rpm in three 30 s cycles with a pause of 30 s between cycles. For equilibration, the homogenizer tubes (15 mL) were sonicated for 10 min, shaken (200 rpm) for 40 min at room temperature (analogue orbital shaker 3005, GFL, Burgwedel, Germany), and centrifuged at 4000 rpm (3202 rcf) for 10 min at 4 °C using an Eppendorf centrifuge 5810 R with an A-4-62 rotor (Eppendorf, Hamburg, Germany). The supernatant was subjected to membrane filtration (Chromafil RC-45/15 MS, 0.45 μm, Macherey-Nagel, Düren, Germany), collected in LC–MS vials (1.5 mL, amber, Macherey-Nagel), and analyzed by UHPLC–MS/MS.

### Ultrahigh-Performance Liquid Chromatography–Electrospray Ionization–Tandem Mass Spectrometry (UHPLC–ESI–MS/MS)

UHPLC separation was performed using a Nexera UHPLC system (Shimadzu, Duisburg, Germany) consisting of two LC-40D X3 pump modules, a DGU-405 degasser, an SIL-40C X3 autosampler set at 10 °C, a CTO-40C column oven, and an SCL-40 system controller. System control and data acquisition were performed using Analyst 1.7.3 software (Sciex, Darmstadt, Germany). Chromatography was conducted with 1 μL of each sample on an Acquity UPLC BEH phenyl column (150 × 2.1 mm, 1.7 μm, 100 Å, Waters, Manchester, UK) equipped with an upstream VanGuard UPLC BEH phenyl precolumn (2.1 × 5 mm, 1.7 μm, Waters) at a flow rate of 0.4 mL/min and a column temperature of 45 °C. Solvent A consisting of 0.1% formic acid in H_2_O (v/v) and solvent B comprising 0.1% formic acid in acetonitrile (v/v) were used, and the following gradient was applied: 10% B for 1 min; increased to 20% B in 2 min and 25% B in 1 min; held at 25% B for 1 min; increased to 27% B in 1 min, 30% B in 3 min, and 32% B in 1 min; held at 32% B for 2 min; increased to 35% B in 2.5 min, 37% B in 4.5 min, and 50% B in 3 min; held at 50% B for 1 min; increased to 70% B in 2 min, 75% B in 3 min, and 100% B in 2 min; held at 100% B for 2 min; decreased to 10% B in 3 min; and held at 10% B for 1 min.

#### Tandem Mass Spectrometry (MS/MS)

To quantify (poly)­phenols in L. japonicus extracts, a QTRAP 6500+ mass spectrometer (Sciex, Darmstadt, Germany) with an electrospray ionization probe was employed, operating in the multiple reaction monitoring (MRM) mode and switching between positive and negative ionization modes (ESI±) with an ion-spray voltage of ±4000 V. A temperature of 450 °C was applied, and nitrogen was used as a curtain gas (45 psi), with gas 1 (nebulizer gas) set at 55 psi and gas 2 (heater gas) set at 65 psi. For calibration and quantitation, the most intensive MRM transitions of each metabolite were used (quantifier), whereas the most specific transition was used as the qualifier ion. The transitions from pseudomolecular [M + H]^+^ or [M–H]^−^ to fragment ions induced by collision-activated dissociation were set at “Medium”, and an entrance potential of ±10 V was used. Additional MRM parameters of all analytes and internal standards are given in Table S3 (Supporting Information). These parameters were optimized for each analyte by direct infusion (12 μL/min). The UHPLC–MS/MS data were processed using MultiQuant software (v 3.0.3, Sciex).

### Internal Standard Calibration

A stock solution was prepared containing all analytes in a concentration of 50 μmol/L with deviating concentrations for lupinalbin A (**28**, 25 μmol/L), phaseollin (**41**, 25 μmol/L), and lotuschromone (**42**, 12.5 μmol/L) in a mixture of DMSO-*d*_6_ and acetonitrile (8:2, v:v). This stock solution was sequentially diluted 17 times with acetonitrile in the ratio 1:1. To each solution, IS stock solution (2.5 μL) was added to receive a final dilution series measured in technical duplicates for external calibration ranging from 0.00076 to 49.8 μmol/L for **1**–**3**, **5**, **6**, **9**–**12**, **14**–**27**, **29**–**40**, **43**–**49**, and **51**–**55**. The calibration ranges were 0.00038–24.9 μmol/L for **28** and **41** and 0.00019–12.4 μmol/L for **42**. Calibration was achieved by plotting the peak area ratios of each analyte and the corresponding IS (*y*) versus concentration ratios of the analyte and IS (*x*). Using power regression, calibration curves with the common formula (1) shown in Table 1 were generated for each analyte (Table S4 in the Supporting Information). The coefficient of determination (*R* ^2^) was >0.99 for all calibration curves. Calibration limits comprising a precise fit for the calibration curves are given in Table S4 in the Supporting Information.

**Table 1:** Equations for the Power Regression and for the Limit of Detection (LOD) and Limit of Quantification (LOQ) Calculation

$y=ax^{b}$	(1)
regression formula	
$y_{\mathrm{crit}}=y_{\mathrm{blank}}+{\mathrm{SD}}_{\mathrm{blank}}\times t_{\alpha =0.05;f=N-1}\times \sqrt{\frac{1}{m}+\frac{1}{N}}$	(2)
$x_{\mathrm{crit}}={\left(\frac{y_{\mathrm{crit}}}{a}\right)}^{1/b}$	(3)
$\mathrm{LOD}=x_{\mathrm{crit}}\times c_{\mathrm{IS}}$	(4)
LOQ=LOQ×3	(5)
*y*_blank_	mean of noise signal intensity
SD_blank_	standard deviation of noise signal intensity
*t*_α = 0.05;*f* = *N* – 1_	*t*-value
*m*	number of repeated analyses
*N*	number of independent blank samples
*c*_IS_	internal standard concentration

### Validation Experiments

Validation experiments were conducted with mature, non-colonized L. japonicus roots and shoots, as no analyte-free matrix was available. The plants were grown to maturity (10–12 weeks) in a greenhouse. After harvesting, the roots were separated from the shoots, rinsed thoroughly with water, lyophilized, and ground to a homogeneous powder using a Grindomix GM 300 knife mill (Retsch, Haan, Germany). The powder was stored at −21 °C until further use.

#### Limit of Detection (LOD) and Limit of Quantification (LOQ)

Ten (*N*) blank samples of roots and shoots were independently prepared and analyzed in triplicates (*m*) in accordance with DIN 32645 guidelines.^78^ Means (*y*_blank_) and standard deviations (SD_blank_) of noise signals were determined in the matrix for all analytes, and for analytes lacking an analyte-free matrix, noise signals directly next to the analyte signal were integrated and averaged. The calculation of the critical signal intensity *y*_crit_ was based on a one-sided *t*-value with the *P*_α_ error set to 0.05 and a degree of freedom *f* of *N – 1*. To obtain the LOD, the critical concentration ratio *x*_crit_ was derived using *y*_crit_ and the regression formula and then multiplied by the concentration of the IS *c*_IS_ (formulas (2–5) in Table 1). The LOQ was defined as three times the LOD.

#### Analyte Recovery and Precision

Five L. japonicus root and shoot matrix samples were prepared and analyzed as described above to determine analyte concentrations in the matrix. For spiking experiments, the root and shoot matrices were spiked with each analyte at five different concentrations with five replicate samples (*N*) per concentration level (Table S5). Analyte recovery was calculated by dividing the mean of the determined concentration by the calculated concentration. To determine intraday precision (as the coefficient of variation [CV]), the samples of spiking level 3 (*N* = 5) were analyzed five times (*m*) within 1 day. To determine interday precision, this procedure was repeated after 48 h.

#### Lyophilization Stability

As the samples were lyophilized before processing, the stability of the analytes during freeze-drying was assessed. Homogenized root and shoot matrices were weighed and transferred to homogenizer tubes; four samples each were lyophilized and prepared as described above, whereas four samples were prepared without lyophilization. Analyte levels were calculated in fresh weight, and the ratios of analyte concentration in lyophilized and nonlyophilized samples were determined. Analyte concentrations before and after freeze-drying could be calculated only for analytes present in the matrix at levels above the LOQ (Table S6).

### Time-Course Data Analysis

Quantitative data from the time-course experiment were analyzed in R (R environment for statistical computing, version 4.3.2). Multivariate analysis was performed using the PCA function from the package FactoMineR. The ggplot2 package was used to generate principal component analysis (PCA) biplots and trend plots.

## Results and Discussion

In our recent study, untargeted LC–MS profiling indicated that AM symbiosis causes the accumulation of bioactive (poly)­phenols in the roots of the model legume Lotus japonicus.^20^ To assess the (poly)­phenol alterations induced by AM symbiosis more thoroughly, a highly selective and sensitive targeted UHPLC–ESI–MS/MS method for rapid and simultaneous determination and quantification of 50 polyphenolic and phenolic (contextually subsumed as “[poly]­phenols”) metabolites in L. japonicus roots and shoots was developed. Following validation, the method was applied to 254 root and 193 shoot samples of different L. japonicus mutants harvested at different times.

### Method Development

In total, 22 analytes previously identified in L. japonicus were assessed with this method, including catechin (**1**), coumestrol (**16**), lupinalbin A (**28**), ayamenin D (**29**), isoliquiritigenin (**31**), formononetin (**32**), vestitol (**33**), lotuscarpene (**35**), acacetin (**38**), biochanin A (**40**), lotuschromone (**42**), lotusaldehyde (**48**), lupinalbin B (**55**),^20^ epicatechin (**2**),^23^ daidzein (**9**),^21^ myricetin (**6**), quercetin (**12**), kaempferol (**19**), isorhamnetin (**25**),^24^ medicarpin (**36**),^21,28,29^ sativan (**44**),^21,26^ and isosativan (**47**).^25^ Other analytes included the (potential) biosynthetic precursors of **28** (genistein, **17**; 2′-hydroxygenistein, **10**), **33** (2′-hydroxyformononetin, **22**; vestitone, **20**), **38** (apigenin, **18**), **40** (naringenin, **21**; genistein, **17**), and **55** (wighteone, **49**; luteone, **43**). The assessed analytes also included various analyte isomers and derivatives comprising genistin (**3**), sophoricoside (**5**), liquiritigenin (**11**), calycosin (**14**), cladrin (**23**), syringetin (**24**), hesperetin (**26**), 7-hydroxy-5,4′-dimethoxyisoflavone (**27**), mosloflavone (**30**), maackiain (**34**), genkwanin (**37**), salvigenin (**45**), 7,3′,4′-trimethylluteolin (**46**), 5-hydroxy-7,4′-dimethoxyisoflavone (**51**), 3,9-dimethoxycoumestan (**53**), and 5-hydroxy-7,4′-dimethoxyisoflavone (**54**), to encompass a broader analyte or compound class spectrum, and the common legume phytoalexins wedelolactone (**15**),^79^ glyceollin I (**39**),^80^ phaseollin (**41**),^81^ and licoisoflavone B (**52**).^82^ Chemical structures of all analytes and ISs are displayed in Figure 1.

**Figure 1:**
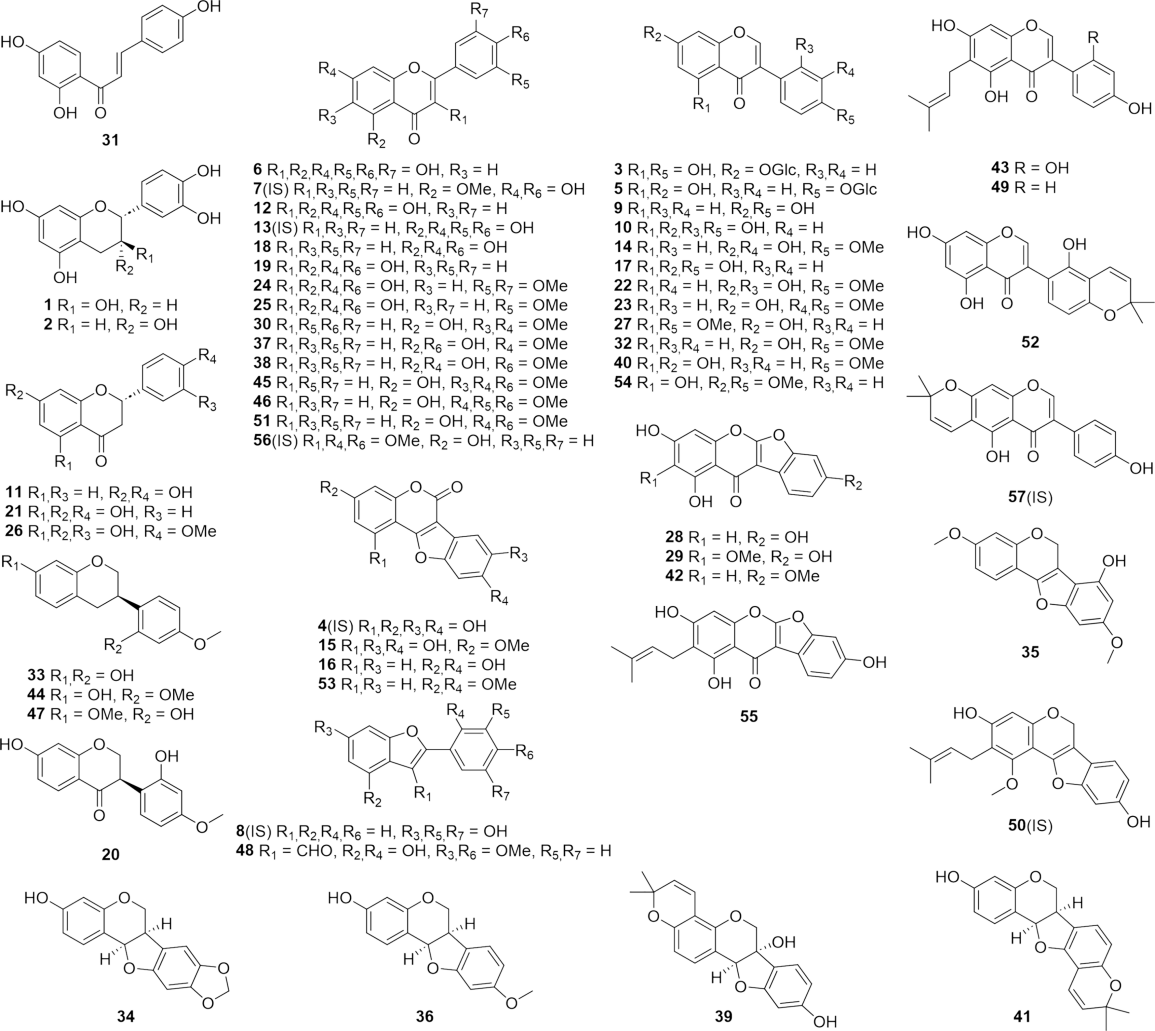
Chemical structures of polyphenol analytes and internal standards (IS) from the ultrahigh-performance liquid chromatography–electrospray ionization–tandem mass spectrometry (UHPLC–ESI–MS/MS) method: catechin (**1**), epicatechin (**2**), genistin (**3**), demethylwedelolactone (**4**; IS), sophoricoside (**5**), myricetin (**6**), thevetiaflavone (**7**; IS), moracin M (**8**; IS), daidzein (**9**), 2′-hydroxygenistein (**10**), liquiritigenin (**11**), quercetin (**12**), luteolin (**13**; IS), calycosin (**14**), wedelolactone (**15**), coumestrol (**16**), genistein (**17**), apigenin (**18**), kaempferol (**19**), vestitone (**20**), naringenin (**21**), 2′-hydroxyformononetin (**22**), cladrin (**23**), syringetin (**24**), isorhamnetin (**25**), hesperetin (**26**), 7-hydroxy-5,4′-dimethoxyisoflavone (**27**), lupinalbin A (**28**), ayamenin D (**29**), mosloflavone (**30**), isoliquiritigenin (**31**), formononetin (**32**), vestitol (**33**), maackiain (**34**), lotuscarpene (**35**), medicarpin (**36**), genkwanin (**37**), acacetin (**38**), glyceollin I (**39**), biochanin A (**40**), phaseollin (**41**), lotuschromone (**42**), luteone (**43**), sativan (**44**), salvigenin (**45**), 7,3′,4′-trimethylluteolin (**46**), isosativan (**47**), lotusaldehyde (**48**), wighteone (**49**), glyurallin A (**50**; IS), 5-hydroxy-7,4′-dimethoxyflavone (**51**), licoisoflavone B (**52**), 3,9-dimethoxycoumestan (**53**), 5-hydroxy-7,4′-dimethoxyisoflavone (**54**), lupinalbin B (**55**), 3,7,4′-trimethylkaempferol (**56**; IS), and alpinumisoflavone (**57**; IS).

Root samples were washed with water after harvesting to remove sand and frozen, resulting in varying amounts of ice being adhered to the frozen samples. Therefore, samples were lyophilized before extraction to determine dry-weight concentrations and enhance reproducibility. Simultaneous sample homogenization and analyte extraction were performed using a tissue homogenizer and acetonitrile as a solvent. For internal calibration, a stock solution containing ISs was added before sample homogenization to compensate for analyte loss during processing. ISs were selected based on common structural features with the analytes, and the L. japonicus root and shoot matrices were screened without ISs to ensure those were not present in the extracts.

LC separation, including MS detection with polarity switching, was achieved on an Acquity UPLC BEH phenyl column (Waters), which ensured efficient resolution (Figure 2A) within a 30 min run time, including a 6 min postrun rinse and equilibration period. Isobaric compounds **11** and **31** with *m*/*z* 254.9; **16** and **32** with *m*/*z* 267.0; **17**/**18** and **36** with *m*/*z* 268.9; **21** and **33** with *m*/*z* 270.9; **7** (IS), **14**, **22**, **28**, **34**, **37**, **38**, and **40** with *m*/*z* 282.9; **10**, **13** (IS), **19**/**20**, **44**, and **47** with *m*/*z* 285.0; **1** and **2** with *m*/*z* 288.9; **23**, **27**, **30**, **35**, **42**, **51**, and **54** with *m*/*z* 299.0 [M + H]^+^ and *m*/*z* 296.9 [M–H]^−^; **12** and **26** with *m*/*z* 300.9; **15**, **29**, and **48** with *m*/*z* 312.9; **45**/**46** and **56** (IS) with *m*/*z* 329.0; **39**, **49**, and **57** (IS) with *m*/*z* 337.0; **50** (IS), **52**, and **55** with *m*/*z* 351.0; and **3** and **5** with *m*/*z* 431.0 were separated chromatographically (see Figure 2B–O). For analytes **1** (*m*/*z* 288.9 → 245.0) and **2** (*m*/*z* 288.9 → 108.9) (Figure 2H), **17** (*m*/*z* 268.9 → 132.8) and **18** (*m*/*z* 268.9 → 116.9) (Figure 2D), **19** (*m*/*z* 285.0 → 185.0) and **20** (*m*/*z* 284.9 → 108.9) (Figure 2G), and **45** (*m*/*z* 329.0 → 268.0) and **46** (*m*/*z* 329.0 → 312.9) (Figure 2L), distinct MRM transitions were specified.

**Figure 2:**
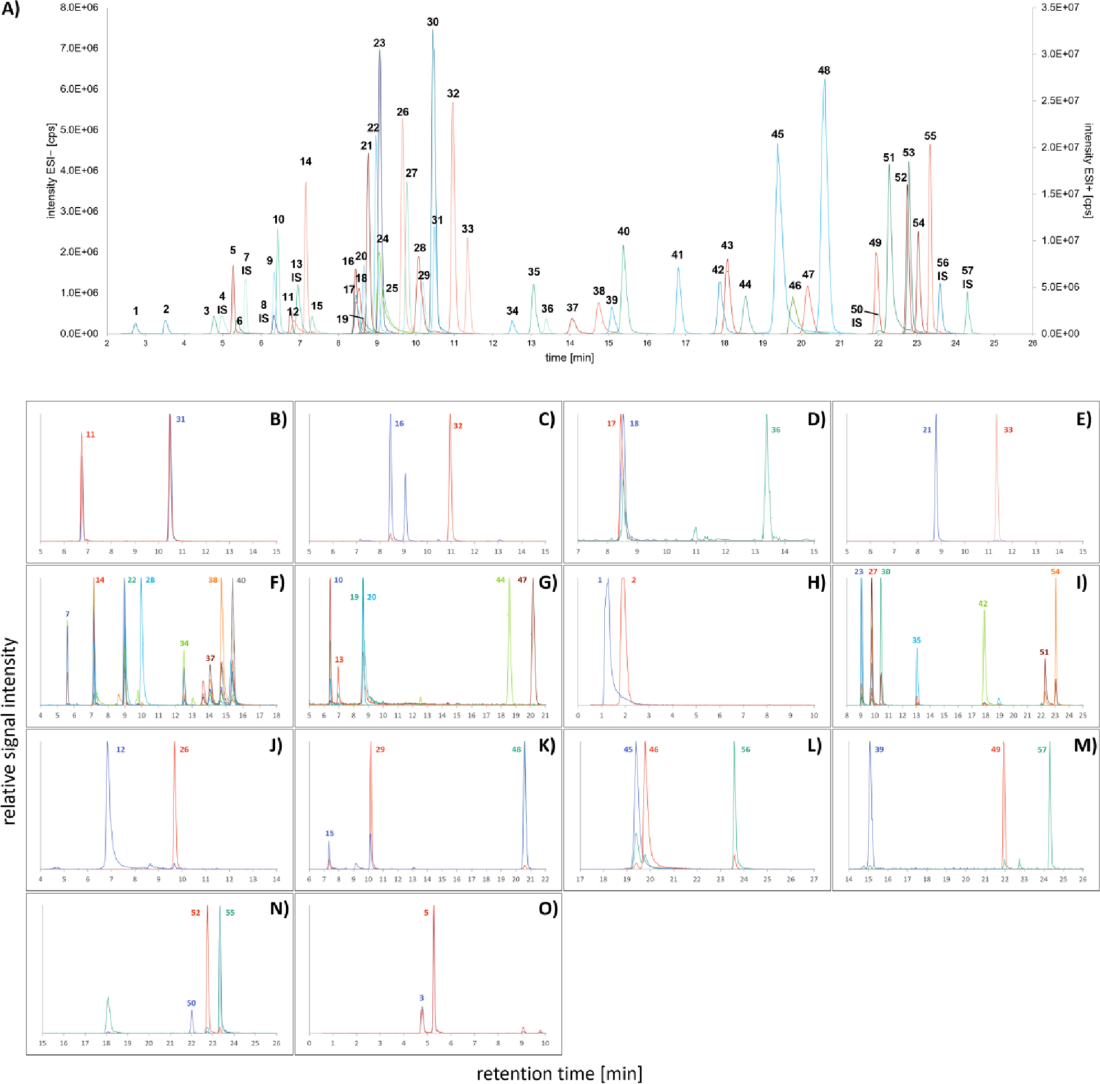
(**A**) Ultrahigh-performance liquid chromatography–electrospray ionization–tandem mass spectrometry (UHPLC–ESI–MS/MS) chromatogram showing quantifier mass transitions in polarity switching mode (ESI±) of distinct polyphenol analytes (*n* = 50) and internal standards (IS; *n* = 7). (B–O) Separation of isobaric analytes in a reference standard mixture. Most isobaric analytes are separated chromatographically except for (H) **1** (*m*/*z* 288.9 → 245.0) and **2** (*m*/*z* 288.9 → 108.9), (D) **17** (*m*/*z* 268.9 → 132.8) and **18** (*m*/*z* 268.9 → 116.9), (G) **19** (*m*/*z* 285.0 → 185.0) and **20** (*m*/*z* 284.9 → 108.9), and (L) **45** (*m*/*z* 329.0 → 268.0) and **46** (*m*/*z* 329.0 → 312.9), which exhibit distinct multiple reaction monitoring (MRM) transitions.

Analytes and ISs were ionized in polarity switching mode (ESI±), and ionization and fragmentation were optimized individually by syringe pump infusion of single standards. Two MRM transitions were selected to identify and quantify analytes, with the most and second most intense and specific transitions used as a quantifier and qualifier, respectively (see Table S3, Supporting Information).

### Method Validation

Calibration was performed in relation to IS concentrations and signal intensities, and the calibration curve was calculated using power functions. The coefficients of determination (*R* ^2^) were >0.99 for all analytes, demonstrating that the chosen functions represented the calibration well within their ranges (see Table S4). LODs ranged from 0.019 nmol/L for **30** to 27.4 nmol/L for **1**. The calibration range varied from 0.012 to 3.1 μmol/L for **6** to 0.8 nmol/L to 24.9 μmol/L for **5**, **14**, **20**, **23**, **27**, **48**, and **49** (Table S4).

Accuracy and precision tests verified the trueness and robustness of the method. Considering the natural occurrence of most analytes in the root and shoot matrices, analyte recovery was assessed by spiking the root and shoot matrices with a reference stock solution at five different concentration levels (given in Table S5) before sample preparation. Recovery rates were highly precise—81–118% for root matrix samples and 81–119% for shoot matrix samples (Table S5)—and were consistent with previously reported polyphenol recovery rates.^83–87^ Natural analyte concentrations in fresh weight of the root and shoot matrices were compared between lyophilized and non-lyophilized samples to assess metabolite robustness preceding sample preparation. Ratios of analyte concentration in the lyophilized and non-lyophilized samples were 85–112% for roots and 86–116% for shoots (see Table S6). Thus, freeze-drying did not affect the analyte concentration. Analysis of intra- and interday precision of the method was performed by repeatedly (*m* = 5) measuring the samples (*N* = 5) of spiking level 3 within 1 day and again after 48 h. The coefficients of variation (CV) were ≤6% (intraday) and ≤17% (interday) in the root matrix and ≤16% (intraday) and ≤15% (interday) in the shoot matrix, confirming intra- and interday repeatability and the applicability of the UHPLC–MS/MS method for quantitative analysis (see Table S5).

### Water Content

To facilitate comparability of dry-weight concentrations, the water content of L. japonicus roots and shoots was determined gravimetrically on the basis of the ratio of sample weight before and after lyophilization. Young (7–10 wpi) L. japonicus roots had a mean water content of 87.1 ± 4.1% (*n* = 64), and shoots had more dry matter with a water content of 72.3 ± 6.1% (*n* = 16). There was no significant difference in the water content of AM and control roots.

### (Poly)­phenol Analysis in Wild-Type and Mutant Lotus japonicus

The (poly)­phenol content in L. japonicus Gifu wild-type roots and shoots was compared with that in two allelic mutants each of *ccamk*, *cyclops*, *ram1*, and *ram2*, which allow no arbuscule formation (*cyclops* and *ccamk*) or only stunted arbuscule formation (*ram1* and *ram2*) in inoculated roots.^69–72^ Roots (7 and 10 wpi) and shoots (10 wpi) with and without R. irregularis inoculation (AM and control, respectively) were compared to investigate the effect of fungal presence on the (poly)­phenol content dependent on the degree of arbuscule formation.

Per sample, two to three roots or shoots from the same pot were pooled to represent one biological replicate (BR), with three BRs in total. AM and control roots were harvested at 7 and 10 wpi, and shoots were harvested at 10 wpi. Samples were lyophilized, mixed with an IS stock solution and acetonitrile, homogenized, and centrifuged, and the supernatants were analyzed. In total, 120 root samples (60 AM and 60 control) and 59 shoot samples (29 AM and 30 control) of wild-type and mutant plants were assayed (Table S2). Analyte concentrations are listed in Table S7 in the Supporting Information.

The (poly)­phenol concentrations in samples of 7 wpi roots, 10 wpi roots, and 10 wpi shoots were analyzed using PCA to visualize the variation in the data set (Figures S1–S4 in the Supporting Information). In all three plots, AM and control mutants were not separated along PC1 and PC2. However, for 7 wpi roots, the wild-type samples showed separation, with control samples arranged along a positive diagonal through quadrant (Q) I and AM samples along a negative diagonal through Q IV (Figure S1). Loading plots revealed that some (poly)­phenols strongly influenced the AM wild-type samples, which were further examined via heat map analysis. PCA separation of 10 wpi wild-type roots was less evident, with AM samples orienting toward a negative diagonal through Q III and IV (Figure S2). Ten wpi mutant root and shoot samples were distributed evenly along PC1 and PC2 (Figure S3).

To examine the concentration differences among samples, (poly)­phenol concentrations were scaled row-wise and displayed as heat maps. Concentrations of compounds **26**, **30**, **34**, **39**, **43**, **45**, **46**, **51**, **52**, and **53** were below LOD or LOQ. Additionally, compounds **10** and **27** were excluded as they were below the LOQ for most of the analyzed samples. All these compounds were included in the group of analytes assessed using the quantitation method but have not been identified in L. japonicus to date. Arithmetic means of analyte concentrations were calculated for wild-type and mutant samples (*ccamk*, *cyclops*, *ram1*, and *ram2*) to differentiate the experimental conditions (roots/shoots, AM/control, 7/10 wpi; cf. Table S7 for single and mean values). Quantitative data were normalized row-wise to perform cluster analysis and depicted as heat maps for roots and shoots (Figure 3A,B). Cluster analysis showed that at 7 wpi, AM wild-type roots were clearly distinguishable from mutant roots (both AM and control) and from AM and control wild-type roots 10 wpi. AM 7 wpi wild-type roots showed the highest levels of myricetin (**6**), calycosin (**14**), wedelolactone (**15**), cladrin (**23**), syringetin (**24**), isorhamnetin (**25**), lupinalbin A (**28**), medicarpin (**36**), acacetin (**38**), biochanin A (**40**), lotuschromone (**42**), isosativan (**47**), and lotusaldehyde (**48**). Those compounds formed a metabolic cluster together with catechin (**1**), lotuscarpene (**35**), and 5-hydroxy-7,4′-dimethoxyisoflavone (**54**), which were also induced in AM 7 wpi wild-type roots. Colonization of wild-type roots also resulted in (slightly) higher levels of quercetin (**12**), kaempferol (**19**), vestitone (**20**), 2′-hydroxyformononetin (**22**), formononetin (**32**), vestitol (**33**), genkwanin (**37**), phaseollin (**41**), sativan (**44**), wighteone (**49**), and lupinalbin B (**55**); however, even higher amounts were detected in some AM and control mutant roots.

**Figure 3:**
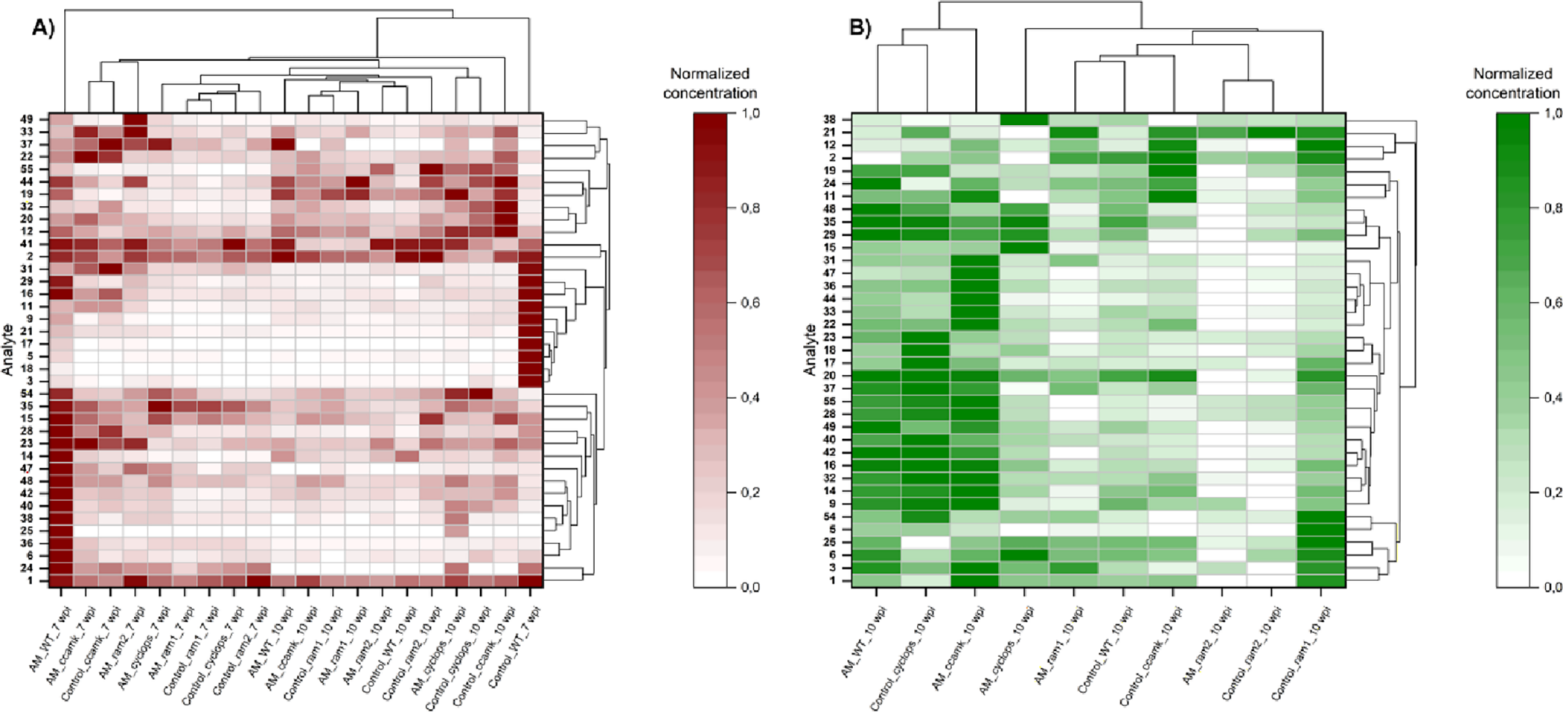
Normalized heat maps of analyte concentrations in wild-type (WT) and mutant (pooled allelic mutants of *ccamk*, *cyclops*, *ram1*, and *ram2*) Lotus japonicus harvested 7 and 10 weeks postinoculation (wpi). Mycorrhizal (AM) and nonmycorrhizal (control) (A) roots and (B) shoots are shown.

The concentrations of catechin (**1**), coumestrol (**16**), and ayamenin D (**29**) did not significantly differ between wild-type 7 wpi AM and control roots. In contrast, the highest concentrations of genistin (**3**), sophoricoside (**5**), daidzein (**9**), liquiritigenin (**11**), genistein (**17**), apigenin (**18**), and naringenin (**21**) and higher concentration of isoliquiritigenin (**31**) were found in control wild-type 7 wpi roots (Figure 3A). These compounds are formed in the early stages of flavonoid and isoflavonoid biosynthesis, with **3** and **5** being the potential storage forms of genistein (**17**), which is a precursor of biochanin A (**40**; cf. the KEGG pathway).^88–90^ Isoliquiritigenin (**31**), liquiritigenin (**11**), and daidzein (**9**) are formed upstream of vestitol (**33**), pterocarpan (e.g., medicarpin, **36**), and coumestan (e.g., coumestrol, **16**) biosynthesis. Naringenin (**21**) is converted to isoflavonoids such as **17** or flavonoids such as apigenin (**18**), which itself is further converted to flavones.^88–90^ Lower amounts of precursor polyphenols and higher amounts of methoxylated (e.g., **14**, **15**, **20**, **22**, **23**, **24**, **25**, **32**, **33**, **36**, **37**, **38**, **40**, **42**, **44**, **47**, **48**, and **54**), prenylated (e.g., **41**, **49** and **55**), or oxidized (e.g., **15**, **28**, **35**, **36**, **41**, **42**, and **48**) compounds indicate an enhancement of the later stages of polyphenol biosynthesis in AM wild-type roots at 7 wpi, that is, during a well-established symbiosis (mean root colonization 65%; Table S7).

Notably, in AM wild-type 10 wpi roots, the concentrations of all analytes in the lower metabolic cluster were lower than those in the 7 wpi roots and sometimes even equal to the levels in the corresponding control roots. Epicatechin (**2**), kaempferol (**19**), genkwanin (**37**), phaseollin (**41**), and sativan (**44**) were present in equal or higher levels in 10 wpi AM roots compared with 7 wpi AM roots. Wild-type 10 wpi control roots showed lower concentrations of early synthesized (poly)­phenols and higher concentrations of late-synthesized (poly)­phenols—as, for example, **2**, **14**, **19**, and **41**—than 7 wpi wild-type control roots (Figure 3A). This observation indicates a decrease in polyphenol biosynthesis in older plants or a shift toward more complex structures, which is discussed later together with the results of the time-course experiment.

Mutant AM and control roots were not distinguishable by polyphenol levels. Cluster analysis revealed that 7 wpi mutant roots (AM and control) were more similar to wild-type AM 7 wpi roots, and 10 wpi mutant roots were more similar to 10 wpi wild-type roots (both AM and control), indicating an age dependency in the regulation of root metabolites. In general, precursor polyphenols, including **3**, **5**, **9**, **11**, **17**, **18**, and **21**, were detected in notably low amounts in both AM and control roots, and more complex (poly)­phenols such as catechin (**1**), epicatechin (**2**), quercetin (**12**), lotuscarpene (**35**), phaseollin (**41**), and 5-hydroxy-7,4′-dimethoxyisoflavone (**54**) were found in concentrations similar to those in AM 7 wpi wild-type roots. In 10 wpi mutants (both AM and control), many analytes were accumulated to levels that exceeded those in 7 and 10 wpi wild-type control roots (e.g., **12**, **19**, **20**, **22**, **32**, **33**, **37**, **44**, **49**, and **55**). For some metabolites, amounts varied strongly in mutant roots, with the overall highest levels observed for, for instance, **33** and **49** in AM *ram2* 7 wpi roots and **12**, **20**, and **32** in control *ccamk* 10 wpi roots (Figure 3A). These results demonstrate that the absence of AM genes interferes with polyphenol biosynthesis in both inoculated and control roots. However, in different mutants, different (poly)­phenols seem to be favored, and no distinct trend toward specific compounds was detected. Previous studies suggested that changes in the flavonoid pattern are linked to different developmental stages of AM formation,^56,57,91^ but based on the analysis of the mutants, we were not able to conclude the same for (poly)­phenols in L. japonicus mutants.

For 10 wpi shoots (Figure 3B), cluster analysis did not show a distinct separation between wild-type and mutants and between AM and control. In wild-type AM shoots, almost all (poly)­phenols were more abundant than in control shoots except some, including **2** and **38**. Notably, a majority of the more complex (poly)­phenols induced in 7 wpi wild-type roots were accumulated in mycorrhizal wild-type shoots at 10 wpi. Variation between AM and control mutant shoots was evident, with higher (poly)­phenol levels in control *cyclops* and AM *ccamk* and lower levels in AM *ram2* and control *ram2*. As observed in roots, the mutations seem to lead to a change in polyphenol biosynthesis in shoots as compared to the wild type, however with no apparent specific trend.

### Time-Course Analysis of (Poly)­phenol Accumulation in Wild-Type Lotus japonicus

To get better insights into the temporal pattern of (poly)­phenol accumulation and AM-induced changes thereof, (poly)­phenols were quantified in AM and control L. japonicus Gifu wild-type roots and shoots, which were harvested at 2, 4, 6, 8, and 10 wpi. AM samples were analyzed individually, with one single root or shoot representing one BR, to account for variability in colonization. Control samples were pooled with three roots or shoots per BR, and all samples were prepared and analyzed as described above. In total, 134 wild-type root (89 AM and 45 control) and 134 wild-type shoot samples (89 AM and 45 control) were analyzed for this experiment (Table S2). All concentrations are given in Table S8 in the Supporting Information.

Root length colonization of AM samples was determined pot-wise, choosing three root systems per pot to give a pot-averaged percentage (Table S8). Over time, an increase in colonization was observed. In 6 wpi roots, the colonization was still below 30%, while at 8 and 10 wpi, colonization was up to maxima of 74 and 85%, respectively. Pot 2 of 8 wpi samples and pot 1 of 10 wpi samples showed noticeably lower colonization compared to the other two pots. By excluding both pots from time-course analysis, the focus was placed on the well-colonized pots to investigate the effect of AM on polyphenol levels.

Compounds **10**, **24**, **26**, **27**, **34**, **39**, **41**, **43**, **45**, **46**, **51**, **52**, and **53** in roots and shoots as well as **25**, **37**, **47**, and **54** in roots and **30** in shoots were below the LOD or LOQ in most or all of the samples and were, therefore, excluded from analysis (Table S8). Time-course trend plots were created to compare the temporal progression of analyte levels in AM and control roots (Figure 4) and shoots (Figure 5).

**Figure 4:**
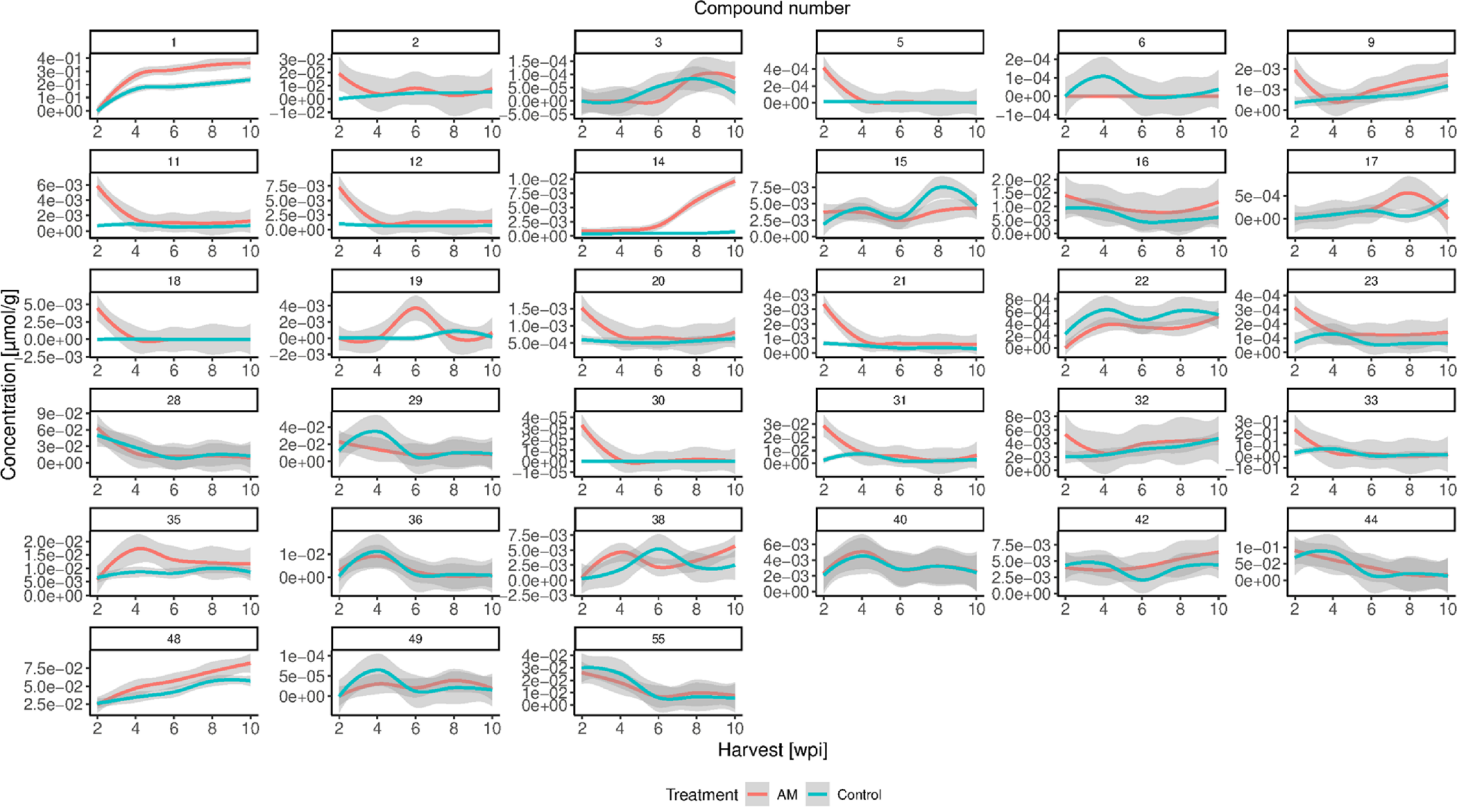
Temporal development of analyte concentrations in AM (red) and control (blue) roots of wild-type (WT) Lotus japonicus after 2, 4, 6, 8, and 10 weeks postinoculation (wpi), including confidence bands (gray).

**Figure 5:**
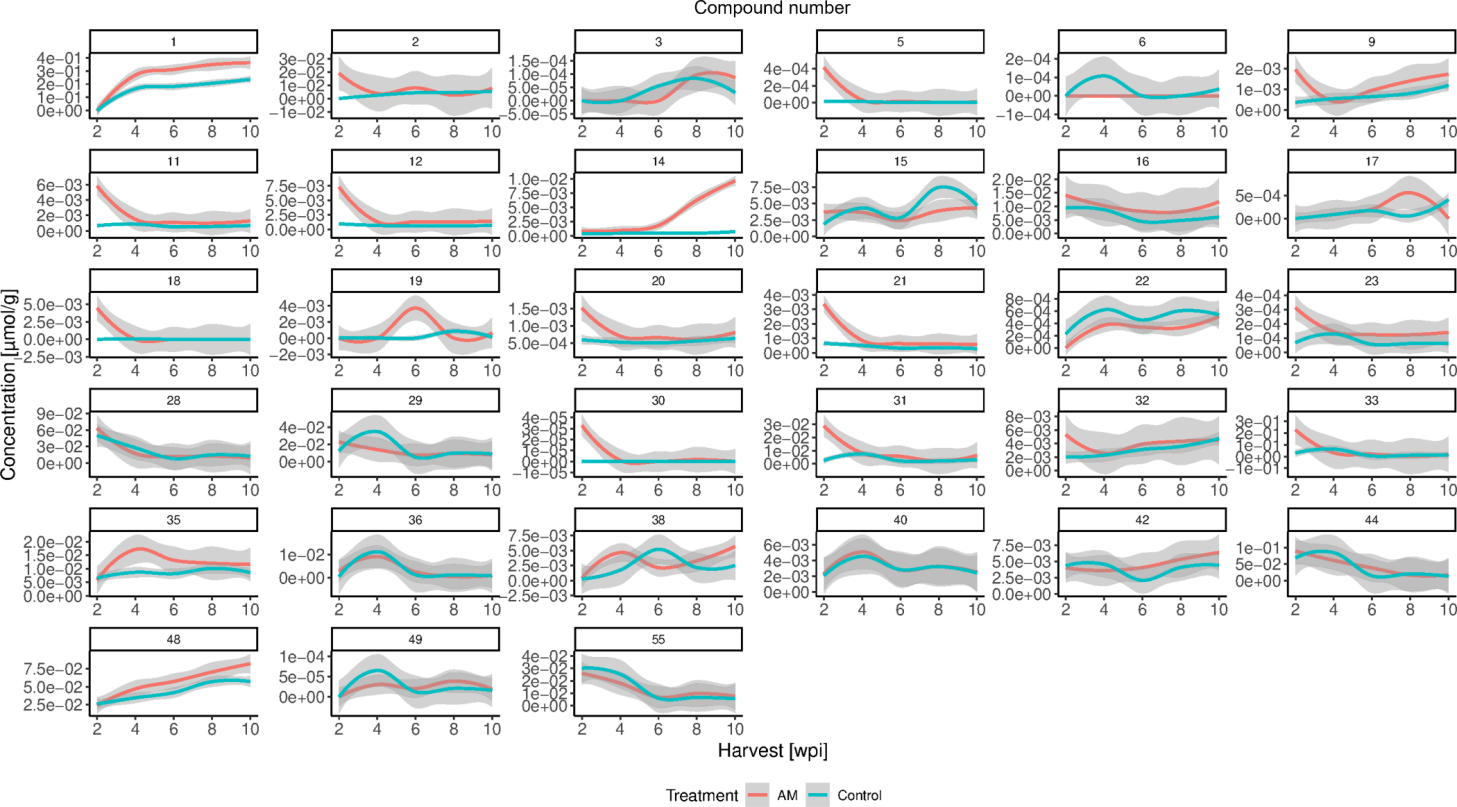
Temporal development of analyte concentrations in AM (red) and control (blue) aboveground organs (shoots) of wild-type (WT) L. japonicus after 2, 4, 6, 8, and 10 weeks post inoculation (wpi), including confidence bands (gray).

In roots, (poly)­phenol concentrations exhibited five different trends: (I) no differences between AM and control roots, including **28**, **29**, **36**, **40**, **44**, and **55**; (II) a mixed trend with concentration fluctuations between AM and control depending on the harvesting time, including **3**, **6**, **15**, **17**, **19**, **38**, and **49**; (III) higher levels in control roots over time, including **22**; (IV) initially higher levels in AM roots with a decline over time, including **2**, **5**, **9**, **11**, **12**, **18**, **20**, **21**, **23**, **30**, **31**, **32**, and **33**; and (V) higher levels in AM roots over time, including **1**, **9**, **14**, **16**, **35**, **42**, and **48** (Figure 4). The same criteria were applied to (poly)­phenols in shoots to group them into five categories: (I) **11**, **14**, **18**, **20**, **21**, **22**, **23**, **28**, **29**, **31**, **33**, and **47**; (II) **16**, **32**, **37**, **38**, **48**, and **54**; (III) **25**; (IV) **1**, **2**, **5**, and **36**; and (V) **3**, **6**, **9**, **12**, **15**, **17**, **19**, **35**, **40**, **42**, **44**, **49**, and **55** (Figure 5).

In general, an increase in (poly)­phenol concentrations in AM roots and shoots was observed compared with control conditions, as only 2′-hydroxyformononetin (**22**) in roots and isorhamnetin (**25**) in shoots were constantly more abundant in control samples (category III). Furthermore, six analytes in roots and 12 analytes in shoots indicated no or only minor variations between AM and control samples (category I), with only **28** and **29** showing equal concentrations between AM and control samples in both roots and shoots. Category II metabolites varied more markedly, without a clear trend separating AM and control lines from each other.

Strikingly, many compounds were highly abundant in young (2 and 4 wpi) AM roots, with a significantly lower concentration in older roots that were equivalent to control levels (category IV). These included some basic (iso)­flavonoids such as daidzein (**9**), liquiritigenin (**11**), apigenin (**18**), vestitone (**20**), naringenin (**21**), isoliquiritigenin (**31**), formononetin (**32**), and vestitol (**33**). Only four polyphenols behaved similarly in shoots, revealing early-stage accumulation as an AM root-specific phenomenon. In general, polyphenol and flavonoid accumulation in AM plants over time is well-reported;^53–57^ however, the specific accumulation in young AM roots suggests a potential polyphenol involvement in the successful initiation of mycorrhizal interactions in L. japonicus. The observed changes in the (poly)­phenolic profile may be linked to root invasion by AMF hyphae at the beginning of symbiosis. Enhanced formation of phytoalexin polyphenolics in roots may regulate plant defense responses involved in AMF invasion.^92,93^

Remarkably, lupinalbins A (**28**) and B (**55**) were found to be distinctly induced under AM conditions in the first experiment (Figure 3A); however, during the time-course experiment, this observation was not reproducible. Similarly, the AM-derived substantial increase in wedelolactone (**15**), kaempferol (**19**), 2′-hydroxyformononetin (**22**), biochanin A (**40**), sativan (**44**), and isosativan (**47**) in roots and calycosin (**14**), coumestrol (**16**), apigenin (**18**), **22**, formononetin (**32**), genkwanin (**37**), **47**, lotusaldehyde (**48**), and 5-hydroxy-7,4′-dimethoxyisoflavone (**54**) in wild-type shoots observed in the first experiment was not detected during temporal monitoring. However, certain metabolites—including catechin (**1**), **14**, and **48** in roots and quercetin (**12**), **15**, genistein (**17**), and **40** in shoots—were consistently more abundant in the AM samples in the time-course experiment. The mutant and time-course experiments were independently conducted over 2 years. Thus, fluctuations in factors such as nutrition and sampling time of the day may have influenced the root metabolome such that different biosynthetic routes were more pronounced in wild-type control roots.

Although seemingly contradictory, these results indicate that AM symbiosis induces an overall accumulation of polyphenols with only a few primary markers common in AM roots in both experiments, such as calycosin (**14**), lotuscarpene (**35**), lotuschromone (**42**), and lotusaldehyde (**48**). In AM shoots, genistin (**3**), kaempferol (**19**), **42**, and **55** were more abundant in both experiments. These results suggest that plant polyphenolics constitute a highly specific blend, which changes with plant age and progression of root colonization by AMF and can be tuned depending on environmental influences. The observation of substantial concentration changes between 2 weeks of sampling, especially in category III metabolites, supports this hypothesis. Previously, a change in the flavonoid pattern of host plants fully colonized by AMF has also been reported.^80,91,94^ Earlier studies attributed polyphenol alterations in mycorrhizal roots to different fungal species,^56^ plant species,^57^ and harvest time and extraction method.^58^ In general, most polyphenolics exhibit similar bioactivities, for example, as phytoalexins or AMF hyphal growth stimulators,^51,67,68,95,96^ but in varying potencies depending on the metabolite concentration and the microbe species involved.^56,60,62^ Additionally, there are contradictory reports of both stimulatory and inihbitory effects of different isoflavonoids and flavonoids on AMF development.^20,59,63–68^ Therefore, polyphenolics are likely to be involved in regulation of AM symbiosis,^56,51^ and the accumulation of specific metabolites may not even be necessary for plants as the desired biological effect could be achieved by generally inducing polyphenol production.^97^

In conclusion, a new highly specific, accurate, and robust UHPLC–MS/MS method was developed and validated to simultaneously quantify 50 (poly)­phenolic analytes, including one chalcone, two flavanols, three flavanones, 12 flavones, three isoflavans, one isoflavanone, 13 isoflavones, two isoflavone glucosides, three coumestans, four pterocarpans, one pterocarp-6a-ene, four coumaronochromones, and one arylbenzofuranaldehyde. The molar concentrations of analytes were determined in the roots and shoots of wild-type and mutant L. japonicus with and without AMF inoculation and in wild-type samples over a 10-week period. A higher overall (poly)­phenol content in mycorrhizal roots and shoots was detected compared with control samples. Mutations in crucial AM genes disrupted polyphenol biosynthesis in such a manner that, across different mutants, single analytes varied markedly, with no clear trend linking one biosynthetic route to a specific gene. During temporal observation over 10 weeks, a shift in root (poly)­phenols was observed. A considerable accumulation of one chalcone, one flavanol, two flavanones, three flavones, one isoflavan, one isoflavanone, three isoflavones, and one isoflavone glucoside was revealed in young (2–4 wpi) AM roots, suggesting a possible involvement in symbiosis initiation. At later stages, different (poly)­phenols were more pronounced in roots and shoots, suggesting that the overall accumulation of polyphenolics may be more important at later stages than the biosynthesis of specific metabolites. These (poly)­phenolics might be involved in negative feedback regulation of symbiosis or they might be associated with the observed increased pathogen resistance of AM roots. Association of genes to biosynthetic pathways and plant mutant analysis will be crucial to understand the importance of individual polyphenols in AM symbiosis. This will provide important information for potentially breeding crops with ideal polyphenol profiles for optimal symbiosis regulation.

## Supplementary Material





## Notes

PCA plots, PC1 and PC2 loadings, and PC variances of 7 and 10 wpi roots and 10 wpi shoots (PDF)
Analyte supplier information, sample list, LC–MS/MS parameters and transitions, values for calibration curve formulas, validation results, and analyte concentrations (XLSX)

## Abbreviations Used

AM: arbuscular mycorrhiza
AMF: arbuscular mycorrhiza fungi
BR: biological replicate
CV: coefficient of variation
DMSO: dimethyl sulfoxide
IS: internal standard
LC–MS: liquid chromatography–mass spectrometry
LOD: limit of detection
LOQ: limit of quantification
MRM: multiple reaction monitoring
PCA: principal component analysis
Q: quadrant
qHNMR: quantitative ^1^H nuclear magnetic resonance spectroscopy
ROS: reactive oxygen species
*R* ^2^: coefficient of determination
SDS: sodium dodecyl sulfate
UHPLC–ESI–MS/MS: ultraperformance liquid chromatography–electrospray ionization–tandem mass spectrometry
wpi: weeks post inoculation
WT: wild-type
