# Supplementary material for: Comprehensive Quantification of (Poly)phenols in Lotus japonicus with and without Arbuscular Mycorrhizal Symbiosis
Source: J Agric Food Chem. 2025 May 26;73(22):14044–57. doi: 10.1021/acs.jafc.5c02061 (PMC12147120; doi:10.1021/acs.jafc.5c02061)

Comprehensive Quantification of (Poly)phenols in *Lotus japonicus* with and without Arbuscular Mycorrhizal Symbiosis

Authors: Josef L. Ranner, Georg Stabl, Cindy Martyniak, Michael Paries, Andrea Spaccasassi, Caroline Gutjahr, Timo D. Stark, Corinna Dawid

Author for correspondence: Corinna Dawid. Email: corinna.dawid@tum.de

The following Supporting Information is available for this article:

Table S1: Analytes and internal standards with corresponding ID numbers and supplier information. (.XLSX)

Table S2: Number of biological replicates and roots/shoots analyzed per replicate. (.XLSX)

Table S3: Retention times, molar masses, multiple reaction monitoring transitions, and MS parameters in negative (ESI−) and positive (ESI+) electrospray ionization mode of analytes and corresponding internal standards. (.XLSX)

Table S4: Values *a* and *b* for the calibration curves with the common formula $y = a \times x^b$, coefficients of determination, limits of detection (LOD) and quantitation (LOQ), and calibration limits. (.XLSX)

Table S5: Analyte recoveries in root and shoot matrix at five different spiking levels and intraday and interday precision of spiking level 3. (.XLSX)

Table S6: Lyophilization stability as ratio of fresh weight analyte concentration in lyophilized and non-lyophilized samples. (.XLSX)

Table S7: Analyte concentrations in mycorrhizal (AM) and non-mycorrhizal (Control) wild-type (WT) and mutant roots and shoots harvested 7 and 10 weeks post-inoculation (wpi). (.XLSX)

Table S8: Analyte concentrations in mycorrhizal (AM) and non-mycorrhizal (Control) wild-type (WT) roots and shoots harvested 2, 4, 6, 8, and 10 weeks post-inoculation (wpi). (.XLSX)

Figure S1: (**A**) Principal component analysis (PCA) and (**B**) PC1 and PC2 loadings denoted as analyte number of 7 wpi inoculated (AM, circles) and non-inoculated (control, triangles) *Lotus japonicus* roots of arbuscular mycorrhizal (AM) mutants (shades of blue) and wild type (WT; red).

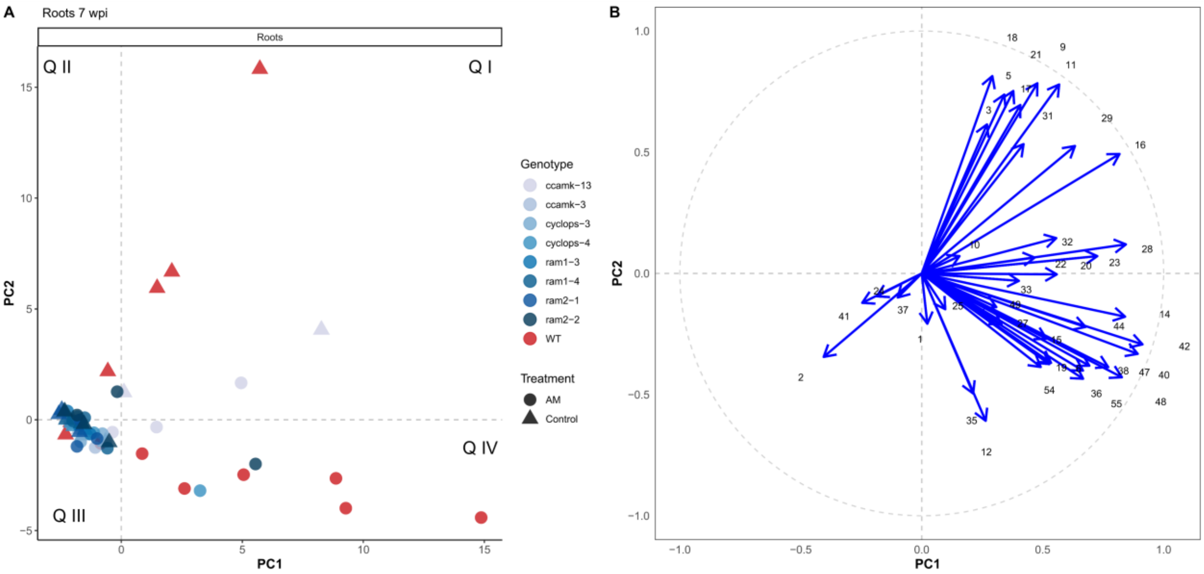

Figure S2: (**A**) Principal component analysis (PCA) and (**B**) PC1 and PC2 loadings denoted as analyte number of 10 wpi inoculated (AM, circles) and non-inoculated (control, triangles) *Lotus japonicus* roots of arbuscular mycorrhizal (AM) mutants (shades of blue) and wild type (WT; red).

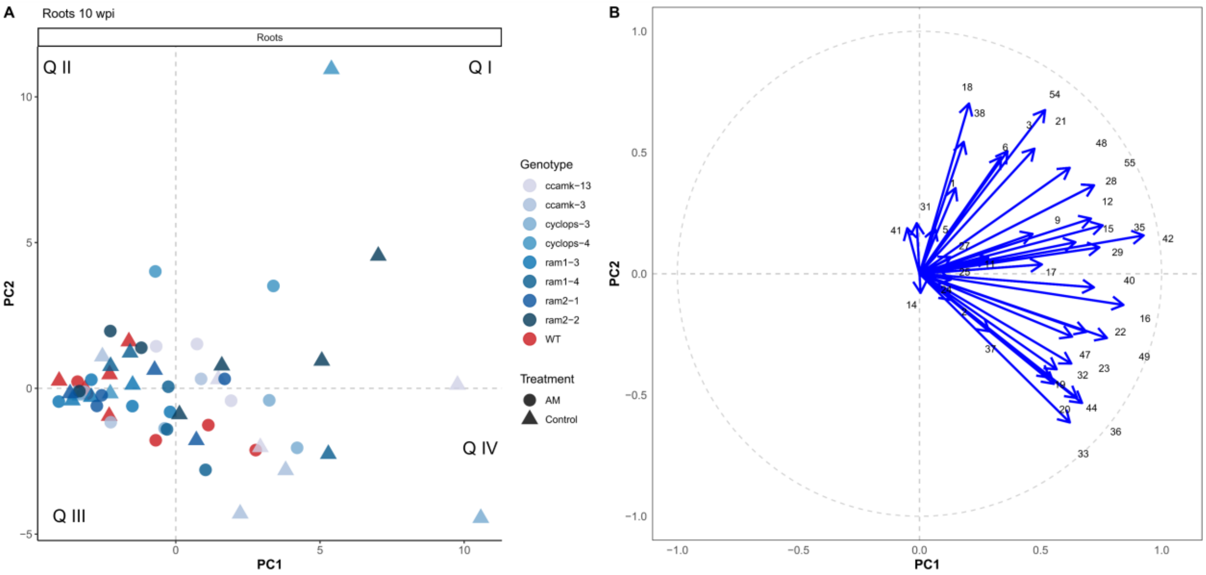

Figure S3: (**A**) Principal component analysis (PCA) and (**B**) PC1 and PC2 loadings denoted as analyte number of 10 wpi inoculated (AM, circles) and non-inoculated (control, triangles) *Lotus japonicus* shoots of arbuscular mycorrhizal (AM) mutants (shades of blue) and wild type (WT; red).

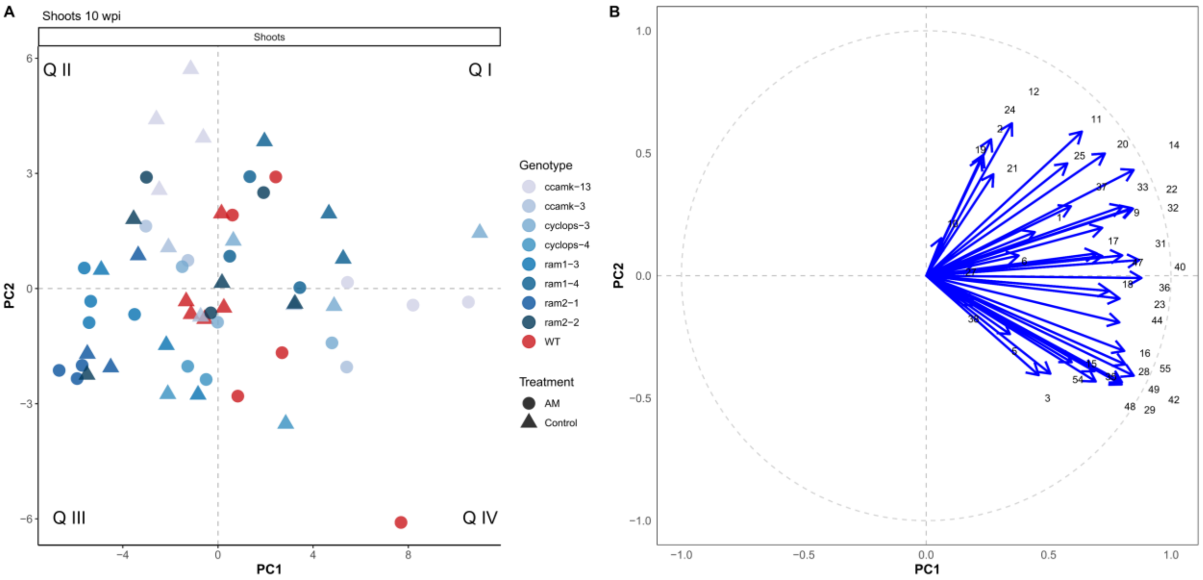

Figure S4: Percentage of variances of the principal components obtained from principal component analysis (PCA) for (**A**) 7 weeks post inoculation (wpi) roots, (**B**) 10 wpi roots, and (**C**) 10 wpi shoots.

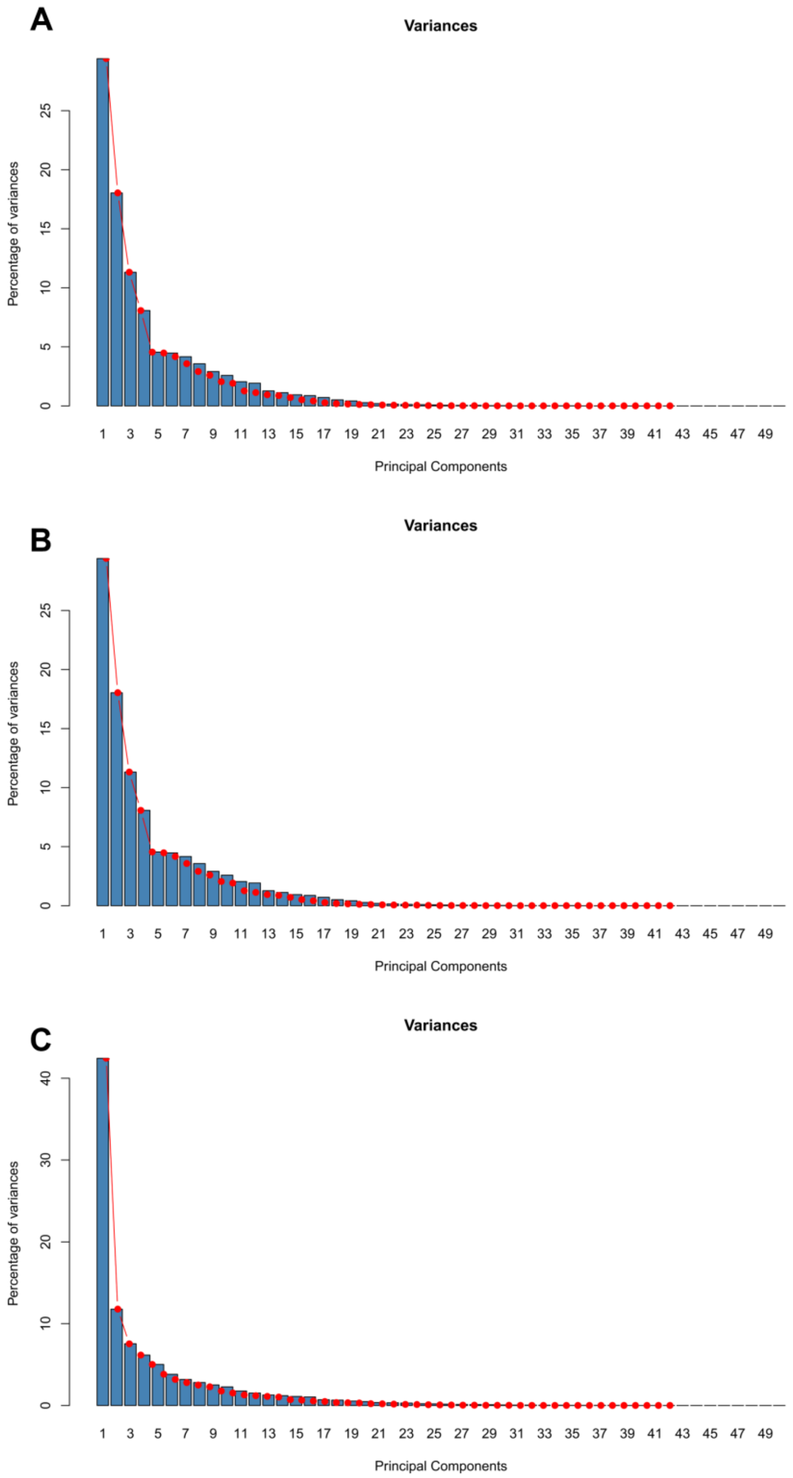

Supplement: Supplementary file 1 [file jf5c02061_si_001.pdf]
